# circATP2B1 Promotes Aerobic Glycolysis in Gastric Cancer Cells Through Regulation of the miR-326 Gene Cluster

**DOI:** 10.3389/fonc.2021.628624

**Published:** 2021-04-15

**Authors:** Xihe Zhao, Zhong Tian, Lei Liu

**Affiliations:** ^1^ Department of Clinical Oncology, Shengjing Hospital of China Medical University, Shenyang, China; ^2^ Department of General Surgery, Shengjing Hospital of China Medical University, Shenyang, China

**Keywords:** gastric cancer, aerobic glycolysis, circATP2B1, miR-326-3p, miR-330-5p, PKM2

## Abstract

The discovery of circular RNA (circRNA) enormously complimented the repertoire of traditional gene expression theory. As a type of endogenous noncoding RNA, circRNA participates in the occurrence of many kinds of tumors in addition to regulating their development. The Warburg effect (aerobic glycolysis is taken with priority for cancer cells instead of oxidative phosphorylation) is one of the most important factors involved in the excessive proliferation of gastric cancer cells. Our data showed that circRNA circATP2B1 (also called hsa_circ_000826) was overexpressed in gastric cancer tissues instead of linear ATP2B1 mRNA, and it promoted aerobic glycolysis in gastric cancer cells. Bioinformatic Gene Ontology analysis showed that the potential downstream targets of circATP2B1 include the microRNA miR-326 gene cluster (miR-326-3p/miR-330-5p), which is functionally focused on cell growth and metabolic processes. The expressions of miR-326-3p/miR-330-5p were downregulated in gastric cancer, and circATP2B1 functionally targeted miR-326-3p/miR-330-5p in an RNA-induced silencing complex (RISC) dependent manner. Dual-luciferase reporter assays demonstrated that pyruvate kinase M2 (PKM2) was one of the targets of miR-326-3p/miR-330-5p. As a rate-limiting enzyme in the aerobic glycolytic pathway, PKM2 accelerated gastric cancer cells’ glucose uptake and increased cell viability. Taken together, circATP2B1 captured miR-326-3p/miR-330-5p and decreased the suppression of PKM2 by miR-326-3p/miR-330-5p, thus aiding the aerobic glycolysis and proliferation of gastric cancer cells. This study identified a novel molecular pathway in gastric cancer that may provide more targets for reversing cancer metabolic reprogramming, as well as a potential strategy for targeted therapy of gastric cancer.

## Introduction

Gastric cancer is one of the most common gastrointestinal malignancies during which patients display no significant symptoms (1). Gastric cancer has poor sensitivity to chemotherapy and current targeted therapies. The pathogenesis of this type of cancer has been a great challenge in recent years, requiring novel approaches for targeted therapies.

Most malignant tumors are characterized by the Warburg effect, an aerobic state in which increased glucose uptake and lactic acid production dominates instead of oxidative phosphorylation to produce ATP in cancer cells (2). In gastric cancer, aerobic glycolysis rates are 200 times as high compared to rates in normal tissues (3). Aerobic glycolysis can induce oncogenesis and promote gastric cancer cell proliferation, as well as mediate resistance to chemotherapy drugs and targeted therapy of gastric cancer. Therefore, the degree of aerobic glycolysis is closely related to the rate of gastric cancer progression.

Our previous work has shown that some noncoding RNAs are involved in glycolysis reprogramming to promote the development of gastric cancer (4). Members of the noncoding RNA class include circular RNAs (circRNAs), which are closed circular RNA molecules (5). circRNA is conserved in most species, and more specifically in certain tissues and developmental stages (6). circRNA is also rich in cytoplasm and does not have a 5’-terminal cap or a 3’-terminal poly (A) tail, so it cannot be degraded by nucleases and is more stable than linear RNA molecules. The stability, high abundance, and specific expression of circRNA suggest that it might play a role in different pathophysiological stages and is an important genetic material (7).

Based on the published data of the high-throughput database circ2Traits (http://gyanxet-beta.com/circdb/), we found that the expression of circATP2B1 (ID: hsa_circ_000826, also known as hsa_circ_0000426, spliced from the ATP2B1 gene) in gastric cancer tissues was significantly higher than in normal gastric mucosa tissues (*P <*0.001). Therefore, it may be involved in the occurrence and development of gastric cancer.

Two studies published simultaneously in *Nature* in 2013 revealed that circRNA acts as a microRNA (miRNA) molecular sponge (7, 8) and that circRNAs can bind to miRNAs in a sequence-specific manner. miRNAs play a crucial role in the regulation of gene expression and cell function (9), including in tumor metabolism which has been widely reported. miRNAs bind to the 3’-untranslated region (3’-UTR) of mRNA to degrade the target gene or inhibit its protein translation (10, 11).

Due to the high expression and stability of circRNA, it exhibits more prominent competitive activity than linear ceRNA to sequester miRNAs, making this a classic function of circRNA (12).

Based on the base complementary pairing principle and the prediction results according to bioinformatic analysis using the CircInteractome and CircNet databases, we screened 12 miRNAs with potential targeted binding sites. Detection in circATP2B1 overexpressive MKN45 cells showed that the expression of the miR-326 gene cluster (miR-326-3p and miR-330-5p) was significantly reduced.

The miR-326 family functions focus on cellular metabolic processes (13). Bioinformatics analysis predicted that miR-326-3p/miR-330-5p have consistent binding sites and that potential target genes include the pyruvate kinase M2 isoform (PKM2) (14). PKM isomers PKM1 and PKM2 possess opposite functions: PKM1 catalyzes the oxidative phosphorylation process (15) while PKM2 catalyzes the aerobic glycolysis process (16). This makes PKM2 a key rate-limiting enzyme of the aerobic glycolysis pathway (17). The content of PKM2 determined the process of glycolysis and high abundance of PKM2 favorined malignancy. Bcl-6 in T cells can inhibit PKM2 and thus inhibit aerobic glycolysis (18). Additionally, c-Myc can increase the ratio of PKM2/PKM1 to promote aerobic glycolysis and tumorigenesis (19). Because PKM2 is an independent prognostic factor for cancer (20), we searched the bioinformatics database Oncomine (https://www.oncomine.org) and noticed that PKM2 was upregulated in gastric cancer. This might be one reason for overproliferation in gastric cancer.

In this study, we investigated the possible intrinsic relationship between circATP2B1 and the miR-326 gene cluster, and their possible mechanisms of action on the glycolysis of gastric cancer. The ultimate goal of this study was to identify a new, potential molecular target for the treatment of gastric cancer.

## Material And Methods

### Human Gastric Cancer Tissue Specimens

Normal gastric tissues (GT), highly differentiated gastric adenocarcinoma cancer specimens (HDAC) and poorly differentiated gastric adenocarcinoma cancer specimens (PDAC) were collected from patients at the Department of General Surgery of Shengjing Hospital of China Medical University (GT n = 18, HDAC n = 17, PDAC n = 15). GT was obtained from normal gastric perforation surgery. Fresh tissues were sent for pathological diagnosis after surgical removal. The remaining samples were then frozen and stored in liquid nitrogen after pathological diagnosis. Before PCR and western detection, all samples were undergone grinding in liquid nitrogen. The ground powder was kept in −80 °C refrigerator. The experiment has been approved by the ethics committee of Shengjing Hospital of China Medical University.

### Cells Lines and Culture

Gastric cancer cells MKN45 and SGC7901, human embryonic kidney cell line HEK-293T and human gastric mucosa cell line GES-1 were obtained from Shanghai Institutes for Biological Sciences Cell Resource Center. MKN45 and HEK-293T cells were hatched in high-glucose DMEM (Dulbecco’s modified Eagle’s medium) supplemented with 10% FBS (fetal bovine serum, Life Technologies Corporation, Paisley, UK). SGC7901 and GES-1 cells were maintained in RPMI-1640 (Sigma-Aldrich, St. Louis, MO, USA) supplemented with 10% FBS. All cells were incubated in a humidified incubator at 37°C with 5% CO_2_. The medium was refreshed every 48 h.

### Fluorescent *In Situ* Hybridization

For identification of circATP2B1 localization in cells, circATP2B1 probe (5’-AATTACGCTCGCAGAGCTGCGGGCT-3’, green-labeled; Biosense, Guangzhou, China) was designed. Fresh normal GT and gastric cancer tissues slices (6 μm) were treated with PCR-grade Proteinase K (Roche Diagnostics, Mannheim, Germany) and blocked using prehybridization buffer (3% BSA in 4 × saline sodium citrate).

Sections were stained with antidigoxin rhodamine conjugate (1:100; Exon Biotech, Guangzhou, China) at 37 °C for 1 h in dark room. DAPI was used for nuclear staining (Beyotime Institute of Biotechnology, Jiangsu, China). Images were taken under a microscope (Olympus, Tokyo, Japan) at ×400 magnifications.

### Plasmid Construction and Cell Transfection

Plasmids of circATP2B1 were constructed for circATP2B1 silencing and overexpressive transfection using pGPU6/GFP/Neo (GenePharma, Shanghai, China) and PCDH (GENESEED, Guangzhou, China) vectors respectively. Short hairpin RNA (shRNA) of ATP2B1 was designed (GenePharma, Shanghai, China). Their respective empty vectors were constructed containing a non-targeting sequence.

For circATP2B1 overexpression, the specially designed front and back circular frames were synthesized and added to pCDH-CMV-MCS-EF1-copGFP for the circulation of transcripts. The front circular frame contains the endogenous flanking genomic sequence with EcoRI restriction enzyme site, and the back circular frame contains part of the inverted upstream sequence with BamHI site. The cDNA encoding circATP2B1 was amplified using primers 5′-CGGAATTCTGAAATATGCTATCTTACAGATGTGTATATCTCATGATTGAT-3′ and 5′-CGGGATCCTCAAGAAAAAATATATTCACGTGCATTATCCCCTTCTGGAGG-3′. The amplified fragment was cloned to the vector between the two frames. circ-ATP2B1 plasmid contained a front circular frame containing the endogenous flanking genomic sequence and a back circular frame, and the back circular frame contains part of the inverted upstream sequence.

The sequence for shRNA targeting circATP2B1 was as follow: AGGGGATAATGCACATGTGTA.

Transfection was performed at about 80% confluence of cells in 24-well plates. Opti-MEM I and Lipofectamine 3000 Reagents (Cat# L300015, Life Technologies Corporation, Carlsbad, CA, USA) were added into cell plates. The stable transfected cells were cultured and selected by Puromycin (400 ng/ml, Sigma-Aldrich, St. Louis, MO, USA). After approximately one month, stable cell lines were set up. The transfected efficiencies were evaluated with qRT-PCR. MKN45 and SGC7901 cells were transiently transfected with chemically synthesized miR-326-3p/miR-330-5p agomir, miR-326-3p/miR-330-5p agomir blank vector, miR-326-3p/miR-330-5p antagomir, miR-326-3p/miR-330-5p antagomir blank vector (GenePharma, Shanghai, China). After 48 h, the transient transfected cells were selected.

Short hairpin RNA (shRNA) of PKM2 was designed: 5’-CATCTACCACTTGCAATTA-3’, and double-stranded DNA (shRNA) was synthesized (GenePharma, Shanghai, China). The stable transfected cells were cultured in medium contained 400 ng/ml G418 (Sigma-Aldrich, St. Louis, MO, USA).

### Real-Time Quantitative PCR (qRT-PCR)

The expression of circATP2B1 was measured using one-step PrimeScript RT-PCR Kit (RR064A; Takara, Japan) through 7500 Fast PCR System. In addition, RNase-R was utilized to eliminate the influence of linear RNAs and confirm the existence of circATP2B1. One Step SYBR PrimeScript RT-PCR Kit (Cat# RR064A, Takara, Japan) was used to qualify the expressions of ATP2B1 and endogenous housekeeping gene GAPDH using relative quantification (2^−△△Ct^). TaqMan MicroRNA Reverse Transcription kit and TaqMan Universal Master Mix II (Cat# 4440040, Applied Biosystems, Foster City, CA, USA) and TaqMan MicroRNA Reverse Transcription Kit (Cat# PN 4366597, Applied Biosystems, Foster City, CA, USA) were used for the detection of miR-326-3p and miR-330-5p. U6 was used as a housekeeping gene with the 2^−△△Ct^ formula.

circATP2B1 primer (Forward 5’-GGCGACATGGCAAACAACTC-3’, Reverse 5’-ATGCATCTGTGGACCTGAGC-3’).
miR-326-3p primer (Forward 5’-CCTCTGGGCCCTTCC-3’, Reverse 5’-CAGTGCGTGTCGTGGAGT-3’)
miR-330-5p primer (Forward 5’-TCTCTGGGCCTGTGTCT-3’, Reverse 5’-TGTCGTGGAGTCGGC-3’).

### Cell Viability Assay

Cell Counting Kit-8 (CCK8, Cat# C0037, Beyotime Institute of Biotechnology, Jiangsu, China) was performed to test the viability of MKN45 and SGC 7901 cells. Cells were seeded in 96-well plates at a density of 2 ∗ 10^3^ cells/well, and 20 μl of CCK8 was added to each well after 48 h incubation. Optical density value (the absorbance) was evaluated at 450 nm with the SpectraMax M5 microplate reader (Molecular Devices, USA).

For EdU incorporation assay, cells were cultured in medium with 10 μM of EdU each well and incubated for 2 h at 37°C. After fixing with 4% formaldehyde, cells were incubated with a BeyoClick™ EdU Kit (Cat# ST067, Beyotime Institute of Biotechnology, Jiangsu, China), then stained with Hoechst 33342 and visualized using a fluorescence microscope (Olympus, Tokyo, Japan). The EdU incorporation rate was measured using the ratio of EdU positive cells (green cells) to total Hoechst 33342 positive cells (blue cells).

### Western Blot Assay

Cells were lysed using RIPA (Beyotime Institute of Biotechnology) buffer on ice for half an hour and went centrifuged at 17,000×*g* for 45 min at 4°C. The protein concentrations were set by the BCA protein assay kit (Beyotime Institute of Biotechnology). All cell lysates with 40 μg proteins were subjected to SDS-PAGE gels and electrophoretically blotted onto PVDF membranes. Membranes were blocked by TTBS (Tween-Tris-buffered saline) containing 5% non-fat milk for 2 h at room temperature and then incubated with primary antibodies as follows: PKM2 Rabbit Polyclonal Antibody (1:1,000, Cat#: ab137852, Abcam, Cambridge, UK), GLUT1 Rabbit Monoclonal Antibody (Cat# AF1015, 1:1,000, Beyotime Institute of Biotechnology, Jiangsu, China), GLUT3 Rabbit Antibody (Cat# bs-1207R, Bioss, Wuhan, China), LDHA Rabbit Monoclonal Antibody (Cat# AF1660, 1:1,000, Beyotime Institute of Biotechnology, Jiangsu, China), and GAPDH Mouse Monoclonal Antibody (1:5,000, Cat# 60004-1-lg, proteintech, USA). After that, membranes containing protein bands were incubated with HRP-polymerization secondary antibodies for 2 h at room temperature. The blots were visualized with ECL chemiluminescent detection system. The integrated density value (IDV) was calculated with software FluorChem2.0.

### Dual-Luciferase Reporter Assay

The putative miR-326-3p/miR-330-5p target binding sequences and mutant sequences in circ-ATP2B1 were synthesized and cloned into the pmirGLO-promoter vector (Promega, Madison, WI, USA). WT pmirGLO-circATP2B1 (or mutated-type pmirGLO-circATP2B1) reporter plasmid and miR-326-3p/miR-330-5p agomir (or agomir NC) were co-transfected into HEK293T cells. The pmirGLO empty vector was used as the Control group. Eighty percentage confluent HEK 293T cells were transfected with Lipofectamine 3000 reagents (Cat# L300015, Life Technologies). Luciferase and Renilla activity was measured 48 h after transfection by a Dual Luciferase Reporter Assay System (Promega, Madison, WI, USA). The ratio of firefly to Renilla luciferase activity was considered as relative luciferase activity.

To examine whether miR-326-3p/miR-330-5p targeted PKM2, respectively, WT PKM2 3’UTR reporter plasmid (PKM2-WT) and mutated-type PKM2 3’UTR reporter plasmid (PKM2-mut) were constructed with pmirGLO-promoter vector. The transfection approach and measurement were carried on as describing above.

### RNA Co-Immunoprecipitation (RIP) Assay

Crude cell lysate was incubated with RIP buffer. For the preparation of RIP buffer, magnetic beads conjugated with human anti-Ago2 antibody or NC normal mouse IgG was added. Complexes were harvested with Proteinase K and then immunoprecipitated RNA was isolated. The RNA concentration measurement and the RNA quality assessment was conducted by a spectrophotometer (NanoDrop, Thermo Scientific, Waltham, MA, USA). To demonstrate the presence of the binding targets, purified RNAs were collected and tested by qRT-PCR.

### Glucose Consumption and Lactate Production Detection

Cells were seeded in 6-well plates at a density of 10^6^ per well at 37°C for 48 h. Cells were kept in serum-free DMEM. The medium at 0 h was collected as background glucose concentration. As previously report (21), the glucose level in the medium was tested using Glucose Uptake Fluorometric Assay Kit (Catalog ^#^ K666-100, BioVision, Cal, US). Glucose concentration reduction of medium was considered as cellular glucose uptake. Glucose uptake = (background concentration − reading concentration)/protein concentration.

For extracellular lactic acid production measurement, Lactate Colorimetric Assay Kit (Catalog ^#^K627-100, BioVision, Cal, US) was utilized (22). The values were normalized to the corresponding protein amounts.

### Detection of ATP/ADP Ratio

The ratio of free ATP/free ADP was measured with ApoSENSOR ADP/ATP Ratio Assay Kit (Catalog ^#^ K255-200, BioVision, CA, USA) (23). Luminescence was measured by spectrometry (Molecular Devices, Cal, US). Total number of 1 × 10^4^ cells were seeded into the luminometer plate and then hatched with nucleotide releasing buffer. One microliter of ATP Monitoring Enzyme was added. Data of 1 min (data A) and 10 min (data B) were recorded. Then the ADP Converting Enzyme was added and samples values were read (data C). The ATP/ADP ratio = data A/(data C − data B).

### NAD+/NADH Ratio Assay

NAD+/NADH ratio was tested using EnzyChrom™ NAD+/NADH Ratio Assay Kit (Cat# E2ND-100, Bioassay Systems, CA, USA) (24). NAD+ and NADH extraction buffer were used respectively to resuspend cells for NAD+ and NADH determination. 10^5^ cells were collected for detection for each sample and washed by cold water. Homogenize samples were put in a 1.5 ml Eppindorf tube with either 100 ml NAD extraction buffer for NAD determination or 100 ml NADH extraction buffer for NADH determination. After heating at 60°C for 5 min, 20 μl of assay buffer was added into the extracts and 100 ml of the opposite extraction buffer was added to neutralize the extracts. Then made the calibration curve. Then the mixtures went for centrifugation at 13,000 rpm for 5e min. Supernatants were transferred to working reagent and the optical densities at 565 nm were read at 0 and 15 min. Absorbance values were used to calculate NAD+/NADH ratios according to the calibration curve.

### Subcutaneous Xenograft Model in Nude Mice

For the *in vivo* study, the stably transfected cells were used. All animal procedures were performed in accordance to the protocols approved by the Animal Care Committee of the Shengjing Hospital. Four-week-old BALB/C anthemic nude mice were used to establish a xenograft tumor model (National Laboratory Animal Center, Beijing, China).

The nude mice were divided into three groups: control group, circATP2B1(−) group, circ-ATP2B1(−) +PKM2(−) group. Cells of 5 ∗ 10^6^ were injected into the flank of each nude mice. The width and length of the subcutaneous tumor were measured every 3 days. Tumor volumes were calculated as: length ∗ width ∗ width/2.

### Statistical Analysis

All quantitative data were taken from at least three independent experiments. Statistical analysis was performed with Student’s t test, nonparametric Mann Whitney U test. Kaplan–Meier plot using SPSS 18.0 statistical software. The results were shown as means ± SD. It was considered statistically significant when p <0.05.

## Results

### The Identification and Characterization of circATP2B1 in Gastric Cancer

According to the high expression of circATP2B1 in high-throughput database, it might exert tumor progression. To validate the prediction of circATP2B1’s tumor promoting role, we first evaluated circATP2B1 expression levels in normal gastric tissues (GT, n = 18), highly differentiated gastric adenocarcinoma specimens (HDAC, n = 17), poorly differentiated gastric adenocarcinoma specimens (PDAC, n = 15) as well as MKN45 and SGC7901 cell lines. Two sets of primers were designed to confirm the circular structure of circATP2B1. Convergent primers were used to detect linear ATP2B1 mRNA and divergent primers were used to detect circATP2B1.

**Figure 1:**
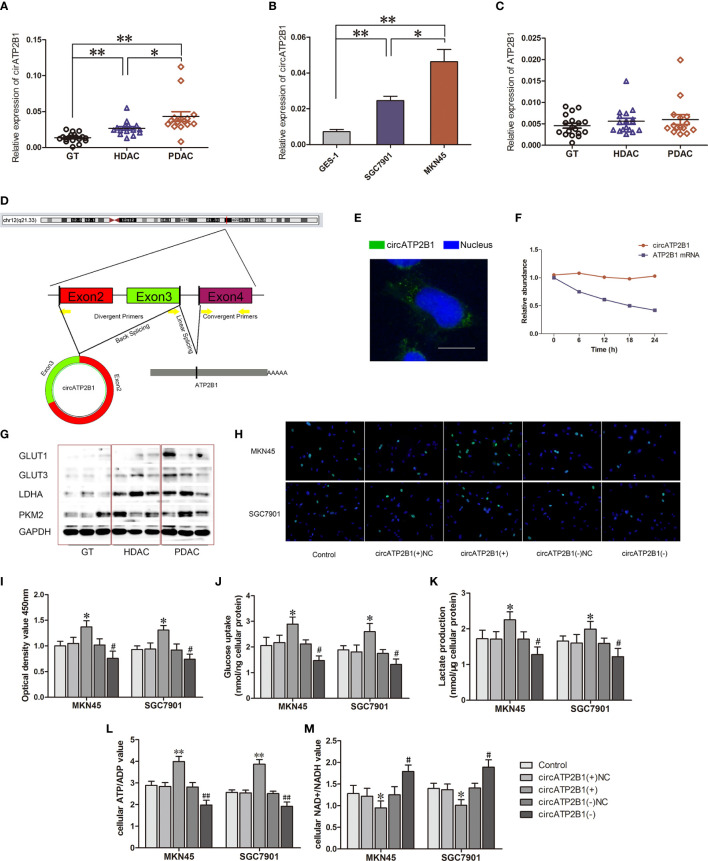
Identification and charactorization of circATP2B1 in gastric cancer tissues and cells. **(A)**. Relative expression of circATP2B1 in normal gastric tissues (GT), highly differentiated adenocarcinoma cancer (HDAC) and poorly differentiated gastric adenocarcinoma cancer (PDAC) specimens. **(B)**. Relative expression of circATP2B1 in gastric cancer cell lines MKN45 and SGC7901 as well as GES-1. **(C)**. Relative expression of ATP2B1 mRNA in GT, HDAC and PDAC specimens. **(D)**. Schematic illustration showing the genomic loci of ATP2B1 gene and circATP2B1 derided from exons 2 and 3 of ATP2B1. **(E)**. Fluorescence hybridization *in situ* (FISH) assay showed the localization of circATP2B1 in cell. The circATP2B1 probe was labeled with FITC (green). Nucleus was stained with DAPI (blue). The image was taken at 1,000× magnification. The scale bar represented 10 μm. **(F)**. Actinomycin assay was performed to evaluate the stability of circATP2B1 and ATP2B1 mRNA in MKN45 cell. **(G)**. Expressions of glycolysis-related proteins GLUT1, GLUT3, LDHA, PKM2 in GT, HDAC and PDAC. **(H)**. EdU incorporation assay and **(I)** CCK8 Cell proliferation assay in circATP2B1(+) and circATP2B1(−) cells. **(J)**. The glucose uptake assay **(K)** lactate accumulation and **(L)** ATP/ADP ratio **(M)** NAD+/NADH ratio of MKN45 and SGC7901 cell lines stably overexpressing or knockdown of circATP2B1. (Data represented means ± SD, n=3, *P < 0.05, **P<0.01, ^#^ *P* < 0.05, ^##^ *P* < 0.01 compared with circATP2B1(-)NC group).

As shown in **Figure 1A**, the existence of circATP2B1 was confirmed through real-time PCR. circATP2B1 expression was significantly higher in PDAC and HDAC than in GT (*P <*0.01 in both groups). In different gastric cancer cell lines, circATP2B1 expression was significantly higher in MKN45 and GSC7901 than in GES-1 cells (*P <*0.01 in both groups, **Figure 1B**). In contrast, there was no significant difference in linear ATP2B1 mRNA expression among GT, HDAC, or PDAC specimens (**Figure 1C**).

The structure of circATP2B1 was investigated using circBase database annotation (**Figure 1D**). circATP2B1 was located at chromosome 12q21.33 (NM_001001323) and was mainly distributed in the cytosol rather than the nucleus, which was demonstrated using *in situ* hybridization (**Figure 1E**). To further analyze the stability of circATP2B1, cells were given actinomycin D to suppress RNA transcription. The results showed that circATP2B1 was more stable following actinomycin D treatment than linear ATP2B1 mRNA (**Figure 1F**). Above all, these results demonstrated that circATP2B1 as a stable non-coding RNA, located in the cytoplasm of gastric cancer cells.

### circATP2B1 Is Positively Associated With Aerobic Glycolysis in Gastric Cancer *In Vitro*

Next, we more closely inspected circATP2B1’s role using gene over-expression and ablation. Our preliminary experiments showed that circATP2B1 exerted gastric cancer cells aerobic glycolysis. We examined glycolytic markers such as Glucose Transporter 1 (GLUT1), Glucose Transporter 3 (GLUT3), L-lactate dehydrogenase A chain (LDHA), pyruvate kinase M2 (PKM2) in GT, HDAC and PDAC. In **Figure 1G**, GLUT1, GLUT3, LDHA and PKM2 showed an increasing trend with the increase of pathological grade. This was consistent with the expression trend of circATP2B1. A small interfering RNA (siRNA) targeting the back-splice junction of circATP2B1 was designed to downregulate circATP2B1’s expression. An overexpression plasmid was also constructed for circATP2B1 without affecting ATP2B1 linear mRNA expression. MKN45 and SGC7901cells overexpressing circATP2B1(+) had increased cell viability, intracellular glucose, lactate production, and ATP/ADP ratios. Furthermore, NAD+/NADH ratios were lower (*P <*0.05, **Figures 1H–M**) in circATP2B1(+) cells compared to control groups. In contrast, the circATP2B1(−) group harbored lower cell viability, intracellular glucose, lactate production, ATP/ADP ratios, and higher NAD+/NADH ratios. Collectively, the findings suggested that circATP2B1 facilitated the glycolysis and proliferation of gastric cancer cells *in vitro*.

### circATP2B1 Downregulates miR-326-3p/miR-330-5p Gene Cluster Expression in Gastric Cancers

**Figure 2:**
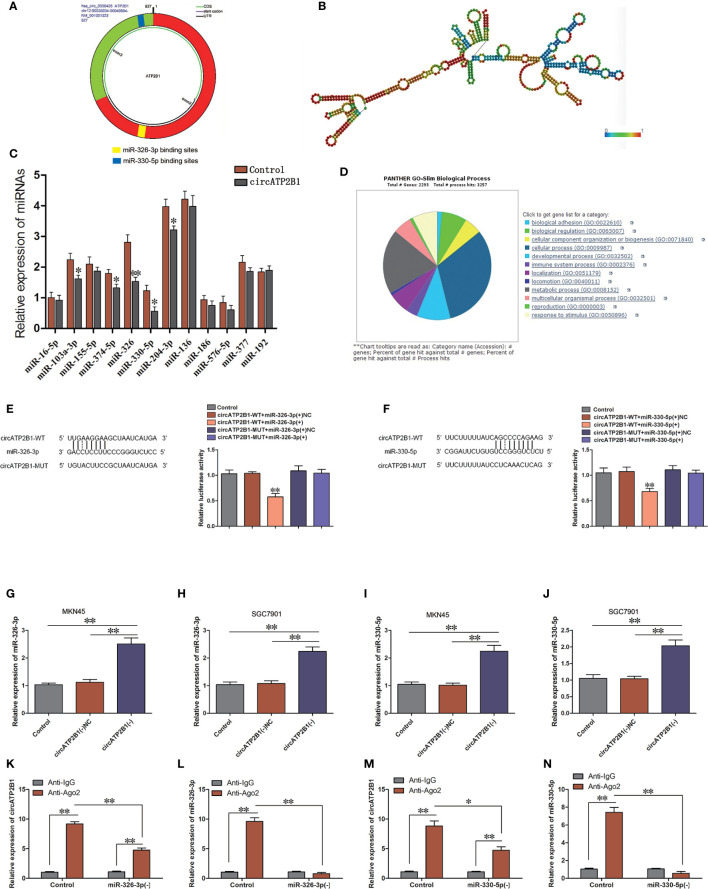
circATP2B1 served as a sponge for miR-326-3p and miR-330-5p in gastric cancer cells. **(A)**. Shematic illustration showing miR-326-3p and miR-330-5p binding sites of circATP2B1. **(B)**. Secondary structure of circATP2B1. **(C–F)** The predicted matching sequence of miR-326-3p **(C)** and miR-330-5p **(E)** to circATP2B1. (circATP2B1-Wt) and the designed mutant sequence (circATP2B1-Mut) were indicated. **(D, F)** Luciferase assay of circATP2B1 and miR-326-3p and miR-330-5p was conducted. Relative luciferase activities for circATP2B1-Wt and circATP2B1-Mut group were presented. Data were presented as mean ± SD. (*P <0.05, **P <0.01). **(G–J)**. Relative expression of miR-326-3p **(G, H)** and miR-330-5p **(I, J)** was investigated in MKN45 and SGC7901 cells using qRT-PCR after circATP2B1 silencing. **(K–N)**. miR-326-3p/miR-330-5p was identified in the circATP2B1-RISC complex. Relative expression of circATP2B1 **(K)**, miR-326-3p **(L)**, circATP2B1 **(M)**, miR-330-5p **(N)** were measured with qRT-PCR. Data represent means ± S D (n = 3 for each group), (*P <0.05, **P <0.01).

A schematic illustration showing potential binding sites for circATP2B1 on miR-326-3p and miR-330-5p is shown in **Figure 2A**. The secondary structure of circATP2B1 is presented in **Figure 2B**. Previous studies have shown that circRNA might serve as a molecular sponge or a ceRNA to miRNA (8). To determine the miRNAs sponged by circATP2B1, 12 candidate miRNAs were predicted using overlapping results from three public databases: TargetScan (http://www.targetscan.org/vert_72/), starBase (http://starbase.sysu.edu.cn/index.php), and miRanda (http://www.microrna.org). Next, we performed real-time PCR in circATP2B1(+) MKN45 cells and circATP2B1(+) negative control cells. Among the miRNAs measured, miR-326 and miR-330-5p showed significantly downregulation in circATP2B1(+) cells (**Figure 2C**). This hinted that circATP2B1 might target miR-326-3p and miR-330-5p using sponging activity. Because miR-326-3p and miR-330-5p are within the same gene cluster, they have similar functions and targets. Gene Ontology (GO) analysis revealed that their functions mainly focused on cellular metabolic pathways (**Figure 2D**). Taken together, the results suggested that circATP2B1 might target miR-326 gene cluster to affect cancer cell metabolism.

### circATP2B1 Functions as a miR-326-3p/miR-330-5p Gene Cluster Sponge in Gastric Cancers

The luciferase reporter assay demonstrated that the relative luciferase activities of pmirGLO-circATP2B1 and miR-326-3p cotransfection were inhibited (*P <*0.01), whereas there was no effect in the miR-326-NC group (negative control) or the mutant construct group (**Figure 2E**). For miR-330-5p, the experiment results were similar. The relative luciferase activities of the circATP2B1-WT + miR-330-5p(+) (treated with agomir) group was downregulated. For the circATP2B1-MUT + miR-330-5p(+) and circATP2B1-WT + miR-330-5p(+) NC groups, the relative luciferase activities were not reduced (**Figure 2F**). This indicates that circATP2B1 directly targets the miR-326 gene cluster.

Subsequently, the expressions of miR-326-3p and miR-330-5p were measured after knockdown of circATP2B1 in MKN45 and SGC7901 cells (**Figures 2G–J**). miR-326-3p and miR-330-5p abundance was significantly increased in circATP2B1(−) groups. Furthermore, an RNA binding immunoprecipitation assay was carried out to detect the involvement of circATP2B1 and the miR-326 gene cluster in the expected RNA induced silencing complex (RISC). The relative expressions of circATP2B1 and miR-326 were increased in the anti-Ago2 groups compared with the anti-IgG groups (**Figures 2K–N**). Taken together, the results indicated that circATP2B1 functioned as a miR-326 gene cluster sponge.

### miR-326-3p/miR-330-5p Trigger Aerobic Glycolysis in Gastric Cancer Cells

**Figure 3:**
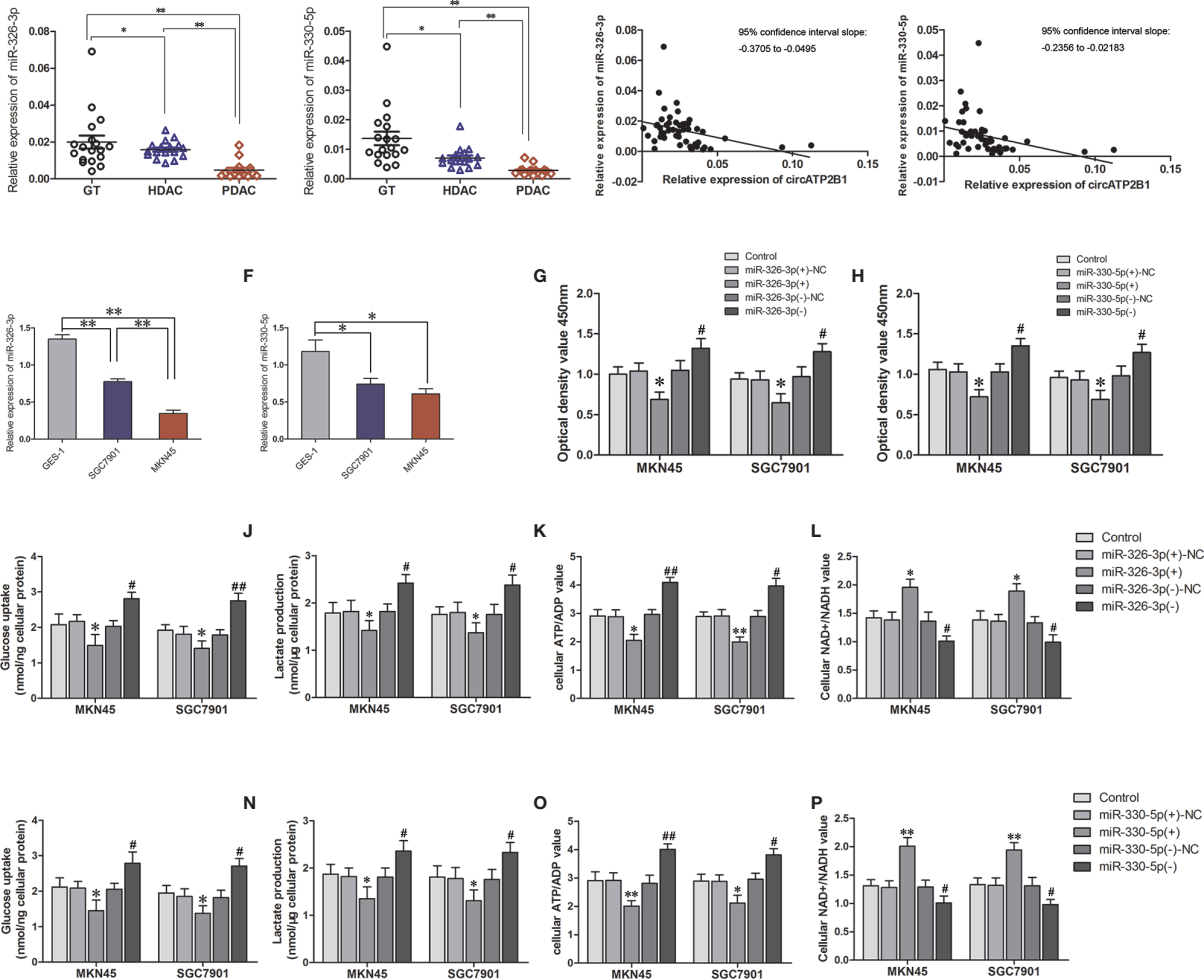
The assuming targets miR-326-3p and miR-330-5p promoted aerpbic glycolysis in MKN45 and SGC7901 cells. **(A, B)**. Relative expressions of miR-326-3p and miR-330-5p in GT, HDAC, PDAC. **(C, D)** Corelationship of circATP2B1 with miR-326-3p and miR-330-5p in GT, HDAC, PDAC. **(E, F)**. Relative expressions of miR-326-3p and miR-330-5p in MKN45 and SGC7901 cells. **(G, H)**. CCK8 assays were performed to evaluate cell viability in MKN45 and SGC7901 cells overexpressing or silence of miR-326-3p or miR-330-5p. **(I)** The glucose uptake assay **(J)** lactate accumulation, **(K)** ATP/ADP ratio. **(L)** NAD+/NADH ratio of MKN45 and SGC7901 cell lines stably overexpressing or silence of miR-326-3p (Data represented means±SD, n=3, *P<0.05, **P < 0.01 compared with miR-330-5p(+)NC group; ^#^ *P* < 0.05, ^##^ *P* < 0.01 compared with miR-330-5p(-)NC group). **(M)** The glucose uptake assay **(N)** lactate accumulation, **(O)** ATP/ADP ratio. **(P)** NAD+/NADH ratio of MKN45 and SGC7901 cell lines stably overexpressing or silence of miR-330-5p. (Data represented means±SD, n=3, *P<0.05, **P < 0.01 compared with miR-330-5p(+)NC group; ^#^ *P* < 0.05, ^##^ *P* < 0.01 compared with miR-330-5p(-)NC group).

In order to detect the basic expression of miR-326-3p/miR-330-5p, miR-326-3p/miR-330-5p expression in GT, HDAC and PDAC were examined with q-RT PCR (**Figures 3A, B**). Contrary to circATP2B1, miR-326-3p/miR-330-5p abundance decreased when pathological grade increased. Correlation analysis showed that miR-326-3p/miR-330-5p expressions were negatively correlated with circATP2B1 (**Figures 3C, D** **)**. The gene abundance of miR-326-3p/miR-330-5p was also detected in cell lines GES-1, MKN45, and SGC7901. The results demonstrated that miR-326-3p and miR-330-5p were significantly downregulated in MKN45 and SGC7901 cells compared to normal gastric cell line GES-1 (**Figures 3E, F** **)**. This coincides with a previous study that showed that the miR-326 gene cluster has an anti-oncogenic role (25).

We then assessed cell viability, intracellular glucose, lactate production, ATP/ADP ratios, and NAD+/NADH ratios in agomir-miR-326-3p/agomir-miR-330-5p and in antagomir-miR-326-3p/antagomir-miR-330-5p gastric cancer cells. For miR-326-3p/miR-330-5p overexpressing cells, cell proliferation was elevated. Glucose uptake, lactate production, and ATP/ADP ratios were also increased, whereas NAD+/NADH ratios was decreased in miR-326-3p(+) or miR-330-5p(+) groups compared to negative control groups, respectively (all of these measures *P <*0.05). Moreover, miR-326-3p or miR-330-5p silencing reduced MKN45 and SGC7901 cell viabilities, glucose uptake, lactate production, and ATP production. Meanwhile, the NAD+/NADH ratios were increased (**Figures 3G–P**). These findings indicated that miR-326 gene cluster promoted gastric cancer cells aerobic glycolysis.

### circATP2B1 Antagonizes miR-326-3p/miR-330-5p Mediated Aerobic Glycolysis

**Figure 4:**
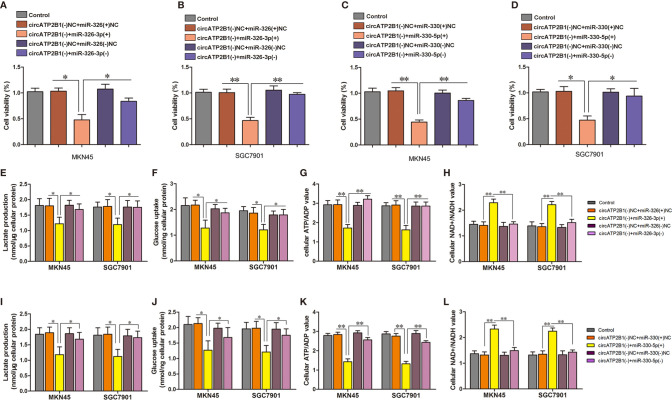
circATP2B1 confered anti-glycolytic effects through regulation of miR-326-3p and miR-330-5p in MKN45 and SGC7901 cells. **(A–D)**. CCK8 assays were performed to evaluate cell viability in MKN45 and SGC7901 cells. **(E, I)** The glucose uptake assay **(F, J)** lactate accumulation and **(G, K)** ATP/ADP ratio **(H, L)** NAD+/NADH ratio of MKN45 and SGC7901 cell lines stably overexpressing or silence of circATP2B1 co-transfected with miR-326-3p or miR-330-5p. (Data represented means ± SD, n = 3, *P <0.05, **P <0.01).

As circ ATP2B1 directly targets the mir-326 gene cluster, which is responsible for aerobic glycolysis, circATP2B1 might play a pro-glycolysis role by sponging miR-326. To investigate this possibility, we performed rescue experiments using siRNAs. Cell viabilities normally decreased by circATP2B1 knockdown were rescued by cotransfection of miR-326-3p siRNA in MKN45 and SGC7901 cells (**Figures 4A, B**). Similarly, miR-330-5p silencing also rescued the cell viability decreases due to circATP2B1 knockdown (**Figures 4C, D**). Glycolysis levels, glucose uptake, lactate production, and ATP production levels were also inhibited after circATP2B1 transfection; this inhibition was partially rescued by miR-326-3p or miR-330-5p silencing (**Figures 4E–G, I–K**). NAD+/NADH ratios were also rescued by addition of miR-326-3p or miR-330-5p (**Figures 4H, L**). The findings suggested that circATP2B1 targeted miR-326 gene cluster to play a pro-glycolytic role.

### miR-326-3p/miR-330-5p Suppresses PKM2 Expression

**Figure 5:**
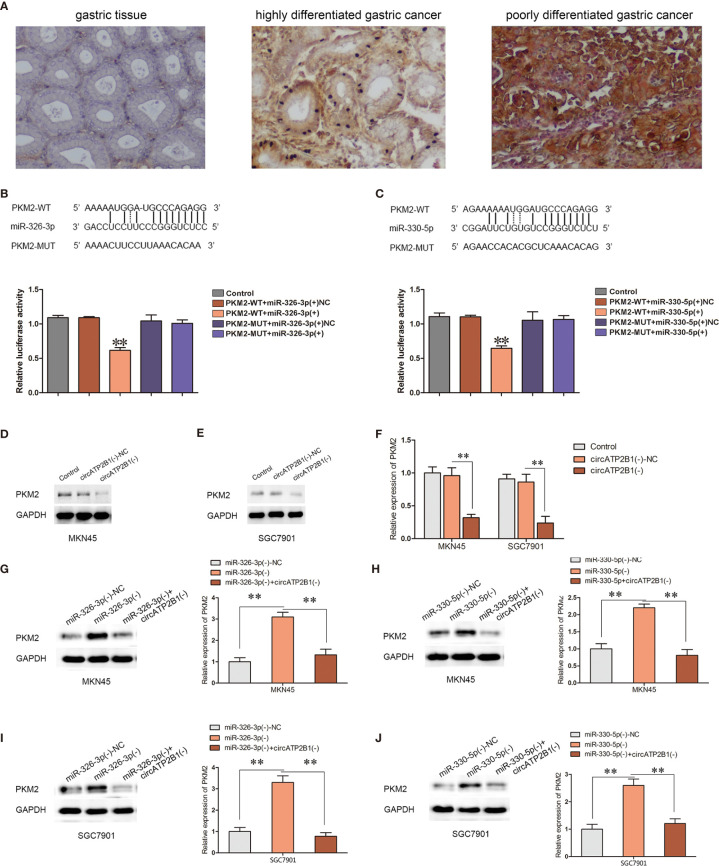
Overexpression of circATP2B1 reversed miR-326-3p/miR-330-5p-induced inhibitory effects on PKM2 protein abundance. **(A)** PKM2 expression in GT, HDAC and PDAC with immunohistochemistry staining. **(B, C)** The predicted miR-326-3p/miR-330-5p binding sites in the 3’-UTR region of PKM2 (PKM2-3’UTR-Wt) and the designed mutant sequence (PKM2-3’UTR-Mut) were indicated. **(F)** Luciferase assay of miR-326-3p/miR-330-5p and PKM2 was conducted. Relative luciferase activities for PKM2-3’UTR-Wt and PKM2-3’UTR-Mut group. **(D–F)**. PKM2 expression change according to circATP2B1 abundance. **(G–J)**. Silencing of circATP2B1 partly reversed miR-326-3p(−)/miR-330-5p(−)-induced elevation on PKM2. Data were presented as the mean ± SD. (*P <0.05, **P <0.01).

Previous studies have described that PKM2, an expected target of the miR-326 gene cluster, has a pro-oncogenic role. As the rate limiting enzyme of glycolysis, PKM2 is a central point of regulation in cancer metabolism. PKM2 expression was investigated in normal gastric tissues, highly differentiated gastric cancer, and poorly differentiated gastric cancer tissues using immunohistochemistry (**Figure 5A**). PKM2 was negative in GT, weakly expressed in HDAC, and strongly expressed in PDAC. A dual luciferase reporter assay was performed to verify the direct targeting of the miR-326 gene cluster to PKM2. The 3’-UTR of PKM2, which contains the putative miR-326-3p or miR-330-5p binding sequences, was cloned into dual-luciferase vectors. The results showed that when the PKM2 wild type plasmid was cotransfected with miR-326-3p or miR-330-5p, the fluorescence intensity decreased significantly (**Figures 5B, C**, P <0.01).

To clarify whether PKM2 is involved in the tumor-promotive role of circATP2B1, the expression levels of PKM2 were assessed in MKN45 and SGC7901 cells. PKM2 expressions were significantly reduced after circATP2B1 silencing (**Figures 5D–F**). In contrast, PKM2 expressions were increased after miR-326-3p or miR-330-5p silencing. Meanwhile, the increase in PKM2 was reduced after circATP2B1 was knocked down in MKN45 and SGC7901 cells (**Figures 5G–J**).

**Figure 6:**
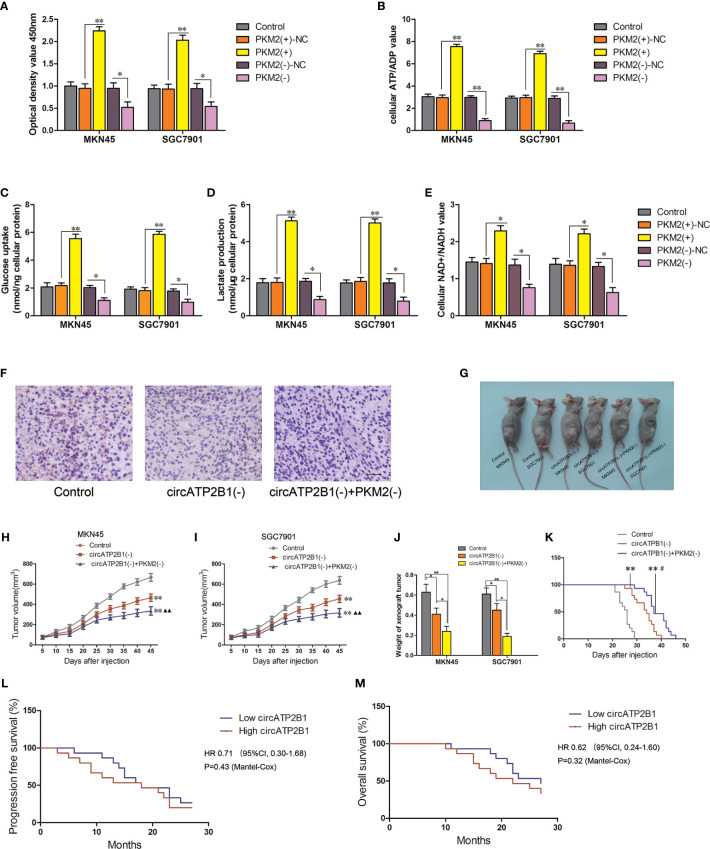
PKM2 knockdown combined with circATP2B1 knockdown play synergistic anti-tumor effect *in vivo*. **(A)**. CCK8 assay conducted for cell viability in PKM2(+) and PKM2(−) groups. **(B)**. The glucose uptake assay **(C)** lactate accumulation and **(D)** ATP/ADP ratio **(E)** NAD+/NADH ratio of MKN45 and SGC7901 cell line which was over-expressing or silencing of PKM2. (*P <0.05, **P <0.01). **(F)**. IHC staining image of ki67 in the subcutaneous xenograft. It revealed the protein level of ki67 in different groups. Images were photographed at 400× magnification. The scale bar represented 50 μm. **(G)**. The nude mice carrying tumors from representative groups were shown (n = 7). **(H, I)**. Tumor growth curves of three groups in nude mice. Tumor growth was monitored for up to 45 days. ^**^ *P <*0.01 vs. control group, ^▲▲^ *P <*0.01 vs.circATP2B1(−) group. **(J)**. Xenogarft tumor weight of three groups in nude mice (*P <0.05, **P <0.01). **(K)**. Kaplan–Meier curves for nude mice xenograft module (**P <0.01 vs Control group, ^#^ *P <*0.05 vs circATP2B1(−) group). **(L)** Progression free survival and **(M)** overall survival for patients with different circATP2B1 expression levels.

The overexpression of PKM2 was reported to relate to oncogenesis in various types of tumors. Immunohistochemistry revealed that PKM2 was highly expressed in gastric cancer cells and its abundance was positively related to pathological grade. This hinted that PKM2 promotes malignant biological behavior in gastric cancer. We then overexpressed and silenced PKM2 in MKN45 and SGC7901 cells. PKM2 overexpression improved cell proliferation, glucose uptake, lactate production, ATP and NADH production, whereas silencing of PKM2 attenuated glycolysis in MKN45 and SGC7901 cells (**Figures 6A–E**). Collectively, miR-326 gene cluster attenuated PKM2 function and supressed glycolysis.

### Knockdown of circATP2B1, Combined With Knockdown of PKM2, Inhibits Its Oncogenic Effects *In Vivo*

Nude mice xenograft models were used to determine whether silencing of circATP2B1 could inhibit cancer growth *in vivo*. In **Figure 6F**, immunohistochemistry showed that, compared with blank MKN45 cells, ki67 expression was weaker in circATP2B1(−) and circATP2B1(−) + PKM2(-) MKN45 cells. As shown in **Figure 6G**, tumor bearing mice of control group, circATP2B1(−) group and circATP2B1(−) + PKM2(−) group were represented (each group had seven mice). Tumor volumes of circATP2B1(−) group were significantly decreased compared with control group in both MKN45 and SGC7901 cells. circATP2B1(−) + PKM2(−) group produced the smallest tumor (**Figures 6H, I** **)**. After the mice were sacrificed, tumor weight measurement was done. circATP2B1(−) group and circATP2B1(−) + PKM2(−) group’s tumor weight was lighter than control group, circATP2B1(−) + PKM2(−) group has the lightest xenograft tumor (**Figure 6J**). Survival analysis of the nude mice models showed that the circATP2B1(−) group and the circATP2B1(−) + PKM2(−) group survived longer after receiving the xenograft compared with the control group (**Figure 6K**, P <0.01 in both groups). Among these, the circATP2B1(−) + PKM2(−) group survived longer than the circATP2B1(−) group (P <0.05). In clinical data analysis, we collected specimens at the Department of General Surgery of Shengjing Hospital of China Medical University (GT n = 18, HDAC n = 17, PDAC n = 15). We then divided the patients into two groups: a high circATP2B1 group and a low circATP2B1 group. Clinical data analysis revealed that patients who had higher circATP2B1 expression had a poorer prognosis. Progression free survival and overall survival of the higher circATP2B1 group was shorter than that of the low circATP2B1 group (**Figures 6L, M** **)**. Our results suggested that circATP2B1 promoted the progression of gastric cancer through miR-326 gene cluster/PKM2 axis.

## Discussion

Gastric cancer is one of the most common cancers worldwide. Although in recent years gastric mortality has declined, gastric cancer still ranks fourth in malignancy incidence and is the third leading cause of cancer-related deaths (26). Despite that diagnosis and treatment methods continue to advance, gastric cancer is still difficult to detect in the early stages. Many patients are diagnosed with gastric cancer in an advanced stage with a poor prognosis. Due to the rapid development of high-throughput sequencing technology, the discovery of circular RNAs in recent years, has attracted the attention of many researchers. circRNA is a class of endogenous noncoding RNA molecules characterized by closed loops without a 5’-terminal cap structure or a 3’-terminal poly A tail. Compared with its homologous linear RNA, circRNA is resistant to RNase digestion due to its closed covalent loops and the absence of sticky ends.

Investigation of the relationship between circular RNA and gastric cancer will help to further clarify gastric cancer occurrence and its development mechanism. It is also expected to provide new ideas in the targeted therapy of gastric cancer. Because of the poor prognosis of gastric cancer and the limitations of current treatment, this study explored abnormal molecular expression during tumor metabolic reprogramming. Until now, only a few circRNAs such as circRNA-100269 (27), circ-KIAA1244 (28), hsa-circ-002059 (29), hsa-circ-0000745 (30), and hsa-circ-0000181 (31) were found to be related to gastric cancer proliferation, differentiation, migration, and apoptosis.

We found that circATP2B1 was significantly increased in gastric adenocarcinoma tissues and gastric cancer cells. This implies that it may play a proto-oncogene role during the oncogenesis and development of gastric cancer. More and more studies are showing that circRNA participates in gastric cancer oncogenesis and progression specifically *via* transcriptional and post-transcriptional regulation (28, 32, 33). Among them, the microRNA sponge mechanism is well studied. circRNA binds miRNA at multiple sites, affecting miRNA activity and thereby regulating the expression levels of miRNA target genes.

The main energy production pattern in normal cells is through oxidative phosphorylation rather than glycolysis. The German scholar Otto Warburg found that glycolysis is also active under aerobic conditions in malignant tumor cells (2). Since tumor cells might preferentially obtain energy through the aerobic glycolysis pathway, which is accompanied by large amounts of lactic acid generation (34, 35), inhibiting glycolysis could promote tumor cell apoptosis and inhibit tumor cell growth (36).

Aberrant cancer energy metabolism patterns have become important targets in anti-tumor treatment (37). Tumor glycolysis can mediate immune resistance to adoptive T cell therapy and impair T cell function (38, 39). By inhibiting glycolysis, researchers can effectively inhibit the proliferation of tumor cells or increase the sensitivity of tumor cells to anti-cancer drugs (40). However, due to the complicated regulatory mechanisms of gastric cancer, different research methods, and sample sizes, the current understanding of gastric cancer-related circRNAs remains controversial and still needs further investigation.

In this study, we found that circATP2B1 regulated the miR-326 gene cluster through a competitive endogenous RNA mechanism. The ceRNA regulation hypothesis has gained substantial attention as a basic post-transcriptional control mechanism for noncoding RNAs such as long noncoding RNAs, pseudogene transcripts, and circRNA (41). Here, miR-326 gene cluster was predicted as a target of circATP2B1. Gene clusters refer to the repetitive units in a gene family, which are closely arranged in large clusters and located in special regions of chromosomes (42). They usually derive from the same ancestor and are composed of two or more copies of a gene through gene duplication (43). They have obvious similarities in structure and function and encode similar protein products. miR-326 is expressed at low levels in gastric cancer and confers an anti-proliferative role (44). The function of the miR-326 gene cluster was enriched in metabolic pathways, according to DAVID enrichment analysis. Furthermore, glioma cells with high levels of PKM2 expressed lower levels of miR-326-3p, suggesting that miR-326-3p endogenously regulates PKM2 (45).

PKM2 has been shown to play a key role in cancer cell metabolism, it binded to β-cantenin in nuclear and boosted tumor growth (46). Variable PKM2 expression and heterozygous PKM2 mutations were found in human tumors suggesting that PKM2 regulation was correlated with different metabolic requirements of cells (14).

Our findings indicate that circATP2B1 overexpression induced metabolic reprogramming, which is a critical oncogenic event in gastric cancer. This provides a strong rationale for targeting circATP2B1 therapeutically in gastric cancer.

## Conclusion

Our study revealed that circATP2B1 can promote the aerobic glycolysis of gastric cancer cells. It competitively binds to miR-326-3p and miR-330-5p, weakening the inhibition of PKM2. As the key enzyme of glycolysis, PKM2 increases glucose catabolism and ATP accumulation; this rapid energy production favors gastric cancer proliferation. Our results suggest that the circATP2B1-miR-326 gene cluster-PKM2 pathway plays an important role in regulating the biological behaviors of gastric cancer cells. Based on these findings, the therapeutic actions of drugs targeting cancer metabolic processes warrant further investigation.

## Data Availability Statement

The raw data supporting the conclusions of this article will be made available by the authors, without undue reservation.

## Ethics Statement

The studies involving human participants were reviewed and approved by the ethics committee of Shengjing Hospital of China Medical University. The patients/participants provided their written informed consent to participate in this study. The animal study was reviewed and approved by the ethics committee of Shengjing Hospital of China Medical University.

## Author Contributions

XZ did nude mice xenografts, wrote and revised the manuscript, and provided funding support. ZT proposed the hypothesis and revised the manuscript. LL did cell culture, transfection, and western assay; he wrote most of the manuscript. All authors contributed to the article and approved the submitted version.

## Funding

This study was funded by National Natural Science Foundation of China (No. 81702402, No. 81802760), Science and Technology Project of Liaoning (No. 20170520027) and 345 talent plan project of Shengjing Hospital (LL, XZ).

## Conflict of Interest

The authors declare that the research was conducted in the absence of any commercial or financial relationships that could be construed as a potential conflict of interest.

## Supplementary Material

Supplementary Table 1 The circATP2B1 sequence.
