# Supplementary material for: circATP2B1 Promotes Aerobic Glycolysis in Gastric Cancer Cells Through Regulation of the miR-326 Gene Cluster
Source: Front Oncol. 2021 Apr 15;11:628624. doi: 10.3389/fonc.2021.628624 (PMC8120303; doi:10.3389/fonc.2021.628624)
Supplement: Supplementary Table 1 — The circATP2B1 sequence. [file Table_1.docx]

circATP2B1 sequence:

ATGTGTATATCTCATGATTGATATGGAGAAACTAGTCATGGGCCAAAGGTCAAGATACTTCTCTGGGAAATGTTGCTGCTGATGCTGCTTTACAAAGTCATACAATGAGTGTTTGGTTTAAGAAAGATTTTCATACTTAAAAGATTTTCATCTTGGAAATACATCAAGTGAAAATTAAATTCTTTTGGGAAACATTTTCCTTCTGATATATTATACTTGTAATGGGCGACATGGCAAACAACTCAGTTGCTTACAGTGGTGTGAAAAACTCTTTGAAGGAAGCTAATCATGATGGAGACTTTGGAATTACGCTCGCAGAGCTGCGGGCTCTCATGGAGCTCAGGTCCACAGATGCATTACGAAAAATACAGGAAAGCTATGGAGATGTCTATGGAATTTGCACCAAATTGAAAACATCTCCCAATGAAGGTTTAAGTGGAAACCCTGCAGATTTAGAAAGAAGAGAAGCAGTGTTTGGAAAGAATTTTATACCTCCTAAAAAGCCAAAAACCTTTCTTCAATTAGTATGGGAAGCATTACAAGATGTCACTTTAATTATATTAGAAATTGCAGCCATAGTATCATTGGGCCTTTCTTTTTATCAGCCTCCAGAAGGGGATAATGCAC
