# Supplementary figures and images for: circATP2B1 Promotes Aerobic Glycolysis in Gastric Cancer Cells Through Regulation of the miR-326 Gene Cluster
Source: Front Oncol. 2021 Apr 15;11:628624. doi: 10.3389/fonc.2021.628624 (PMC8120303; doi:10.3389/fonc.2021.628624)

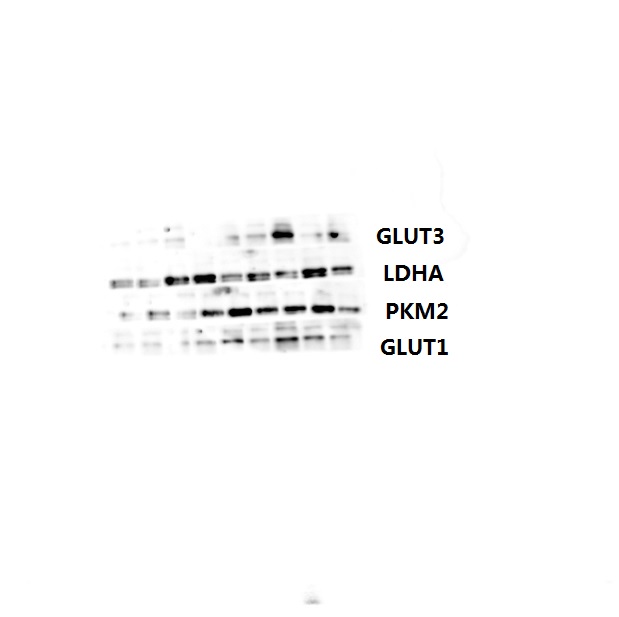

Supplement: Supplementary file 2 [file Image_1.jpeg]

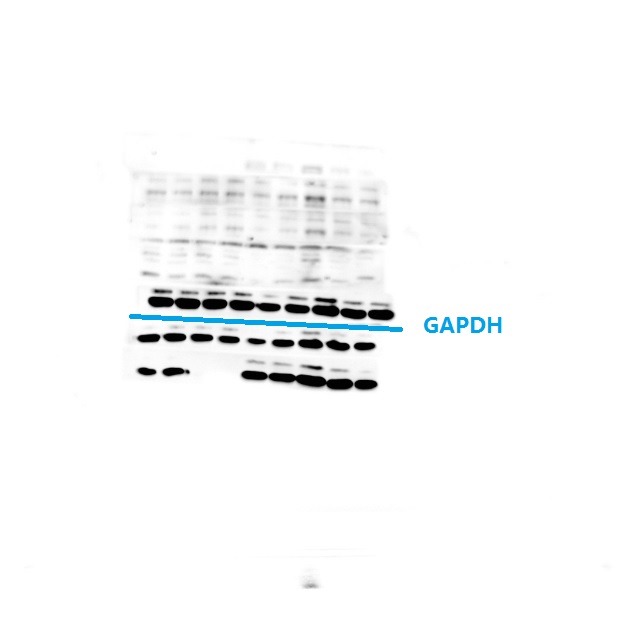

Supplement: Supplementary file 3 [file Image_2.jpeg]

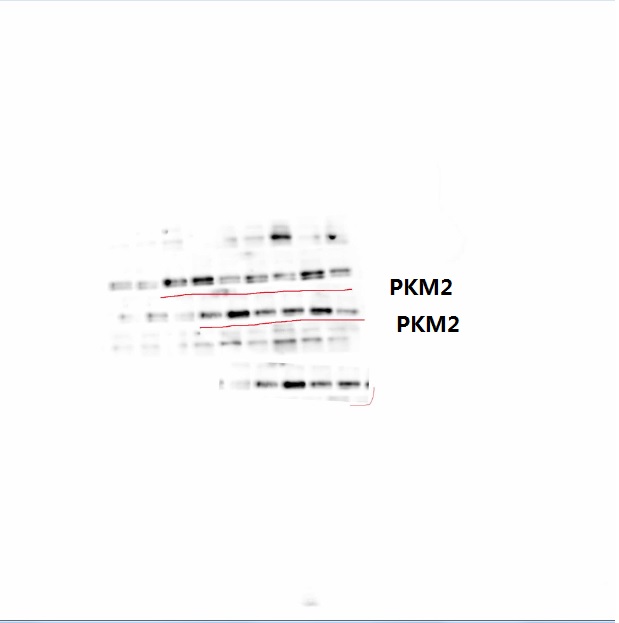

Supplement: Supplementary file 4 [file Image_3.jpeg]

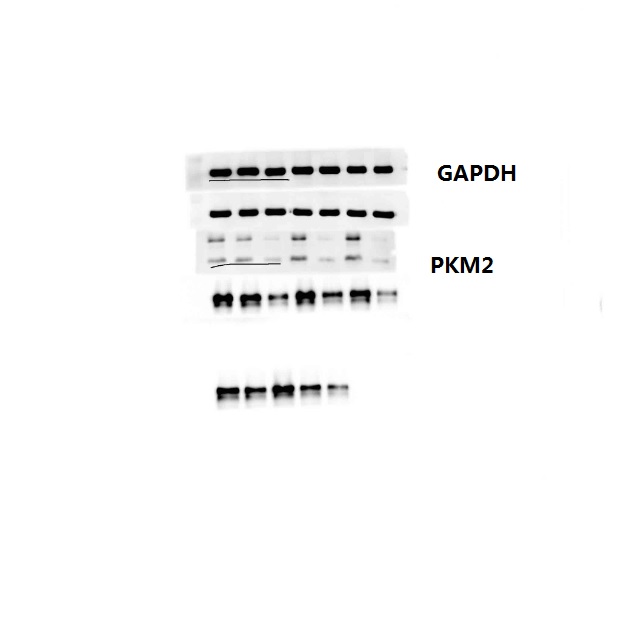

Supplement: Supplementary file 5 [file Image_4.jpeg]

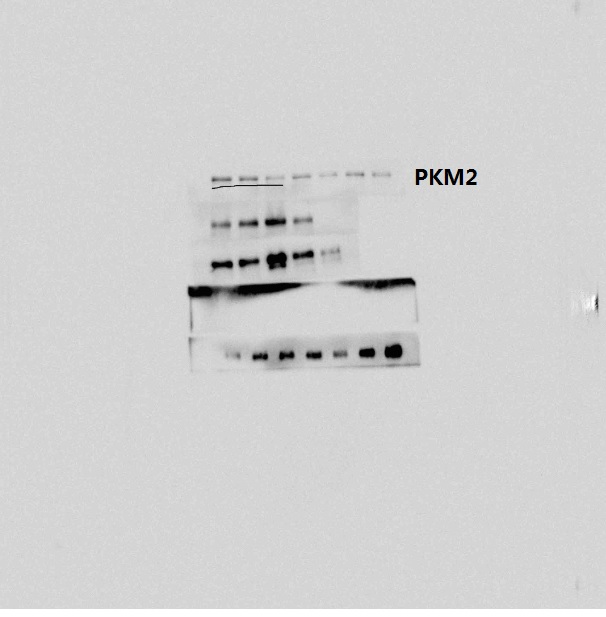

Supplement: Supplementary file 6 [file Image_5.jpeg]

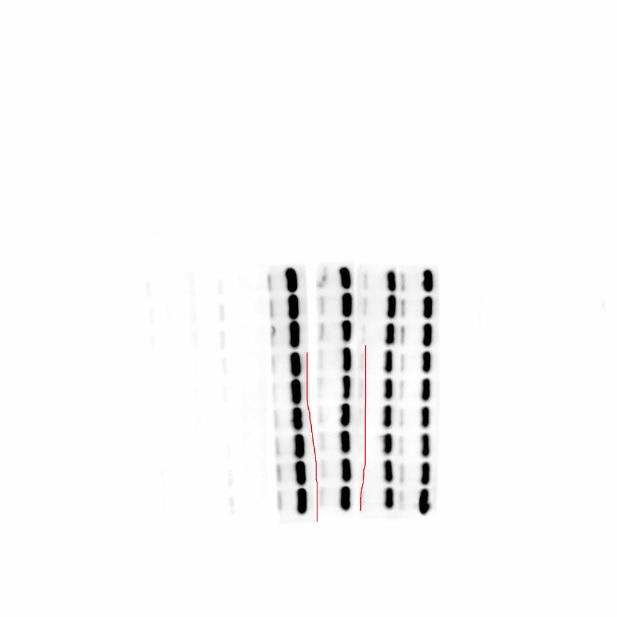

Supplement: Supplementary file 7 [file Image_6.jpeg]
